# Supplementary material for: The role of water molecules in the dissociation of an electron-molecule contact pair
Source: Nat Commun. 2025 Mar 3;16:2113. doi: 10.1038/s41467-025-57403-7 (PMC11876569; doi:10.1038/s41467-025-57403-7)
Supplement: Supplementary file 1 — Supplementary Information [file 41467_2025_57403_MOESM1_ESM.pdf]

Supplementary Information for:

The role of water molecules in the dissociation of an electron-molecule contact pair

Connor J. Clarke[1], E. Michi Burrow[1], Jan R. R. Verlet[1,2]*

[1]*Department of Chemistry, Durham University, Durham DH1 3LE, United Kingdom*

[2]*J. Heyrovský Institute of Physical Chemistry, Czech Academy of Sciences, Dolejškova 3, 18223 Prague 8, Czech Republic*

Email: j.r.r.verlet@durham.ac.uk

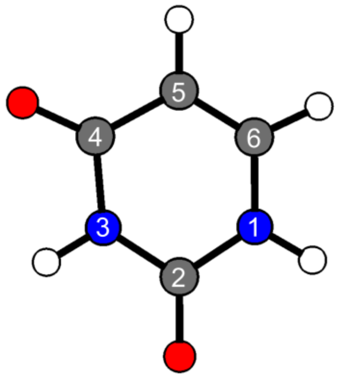

Suppl. Fig. 1. Labelled atoms of the uracil molecule. Colored by atom type: H (white), C (gray), N (blue), O (red).

Suppl. Note 1. Benchmarking the VDE of non-valence anions

In the calculation of non-valence states, it is typically necessary to utilize a custom, extra-diffuse basis set. In line with other similar studies,[1] the aug-cc-pVDZ Dunning basis set[2] was used, appended with additional diffuse functions that where affixed onto the N1 and C6 atoms (see Suppl. Fig. 1), which are located on the positive side of U. Even-tempered (scaling factor of $10^{0.5}$) basis functions were added: m of s character and n of p character, and we denote the basis set aug-cc-pVDZ+msnp. Suppl. Table 1 shows the calculated DFT/CAM-B3LYP[3] electronic energies of U^{-} and neutral U, calculated at the optimized geometry of the anion, with different numbers of diffuse functions added. The difference between these energies is equal to the vertical detachment energy (VDE). Following three s and p functions, the VDE was not found to change enough to justify the computational expense of further diffuse basis functions, and so the aug-cc-pVDZ+3s3p basis set was selected going forward. Suppl. Table 2 displays the exponents and contraction coefficients for the additional (+3s3p) basis functions, formatted to be compatible with a user-specified basis set in Gaussian 16,[4] and with the geometries listed further below.

Suppl. Table 1. DFT/CAM-B3LYP computed electronic energy and vertical detachment energy (VDE) of U^-, using the aug-cc-pVDZ basis set with different numbers of appended even-tempered extra-diffuse basis functions.

Basis set	Energy of U^- / Har	Energy of U / Har	VDE / meV
aug-cc-pVDZ+1s1p	−414.70448	−414.70713	−72.2
aug-cc-pVDZ+2s2p	−414.70993	−414.70716	75.3
aug-cc-pVDZ+3s3p	−414.71110	−414.70717	107.0
aug-cc-pVDZ+4s4p	−414.71130	−414.70717	112.5

Suppl. Table 2. Details of the custom aug-cc-pVDZ+3s3p basis set, formatted for use in Gaussian 16. Atoms numbered '2' and '4' are the N1 and C6 atoms, respectively, as shown in the geometries listed further below.

```
H C N O     0
aug-cc-pvdz
****
2 4 0
S    1   1.00
      0.02345             1.00
S    1   1.00
      0.0074155           1.00
S    1   1.00
      0.002345            1.00
P    1   1.00
      0.020205                1.0000000
P    1   1.00
      0.006389                1.0000000
P    1   1.00
      0.0020205                1.0000000
****
```

In order to assign geometrical structures to each non-valence feature in the photoelectron spectra of the $U^-(H_2O)_n$ anion clusters (Fig. 2b), a two-step procedure was used. The first step performed a relaxed optimization of the cluster structure, and the second step calculated the VDE of the DBS at this optimized geometry. In the first step, density functional theory (DFT) with the long-range corrected CAM-B3LYP functional was used to generate the optimized geometries of the $U^-(H_2O)_n$ anion clusters. DFT was chosen for its high-throughput

capabilities − a wide sample of initial geometries for each cluster could be optimized due to the efficient nature of convergence, leading to an extensive list of possible structures. It should be noted that this list was not exhaustive, but sufficient for satisfactory explanations of the experimental observations. In particular, where water molecules solvated the dipole-bound electron, the potential energy surface along certain water translation coordinates was very shallow, leading to many possible optimized structures. Even in this case, the exact position of the water typically had a negligible effect on the calculated VDE of the DBS.

For more accurate determination of the VDE in the DFT-optimized structure, we performed *ab initio* CCSD(T) calculations.[5,6] To ensure that the electron binding energies of the calculated non-valence states were reliable, we demonstrate some basic benchmarking results for several non-valence states that have experimentally measured electron binding energies (VDEs). The chosen non-valence anions were $(H_2O)_{2-6}^-$, and U^-, providing a wide range of binding energies. Suppl. Table 3 shows the calculated (and experimental) VDEs for the benchmark DBSs, using improving levels of theory. For each non-valence state, the DFT (CAM-B3LYP) energy dramatically overestimated the VDE. Conversely, each *ab initio* method underestimated the experimental VDEs, but became more accurate as higher levels of theory were applied. Ultimately, the VDEs calculated with CCSD(T) theory were accurate to within some 10s of meV for each non-valence anion, and could be considered slight underestimates. We believe our methodology is appropriate for distinguishing between structural isomers that differ in electron binding energies by ~100 meV, as was the case for the measured $U^-(H_2O)_n$ clusters.

Suppl. Table 3. Computed vertical detachment energies (VDEs) of several DBS anions, using different levels of theory and the aug-cc-pVDZ+3s3p basis set. Corresponding experimentally measured values are also shown.

Level of theory	VDE / meV					
	U^-	$(H_2O)_2^-$	$(H_2O)_3^-$	$(H_2O)_4^-$	$(H_2O)_5^-$	$(H_2O)_6^-$
CAM-B3LYP	107.0	126.5	254.7	471.6	482.3	1494
HF	25.9	1.0	68.3	199.1	173.7	225.7
MP2	45.0	21.0	113.4	289.9	282.9	354.8
CCSD	62.9	33.4	139.2	323.8	323.3	396.1
CCSD(T)	68.0	39.1	151.4	344.6	348.4	424.3
Exp.	75 ± 6[a]	45 ± 5[b]	160[c]	350 ± 20[c]	350[c]	480[d]

Taken from [a] 7 and current work, [b] 8, [c] 9, [d] 10. Uncertainties provided where available.

Suppl. Note 2. Uracil-electron and hydrogen bonding distances

Suppl. Fig. 2 displays geometric properties of the all-Q isomers of non-valence $U^-(H_2O)_n$, calculated using the methodology described above. The distance between the centre of mass of the uracil molecule and the point of maximum non-valence electron density (purple arrows) increases with increasing cluster size, demonstrating how the added water molecules drive separation of the uracil-electron contact pair. Hydrogen bonding distances (green arrows) of the clustered water molecules are also shown.

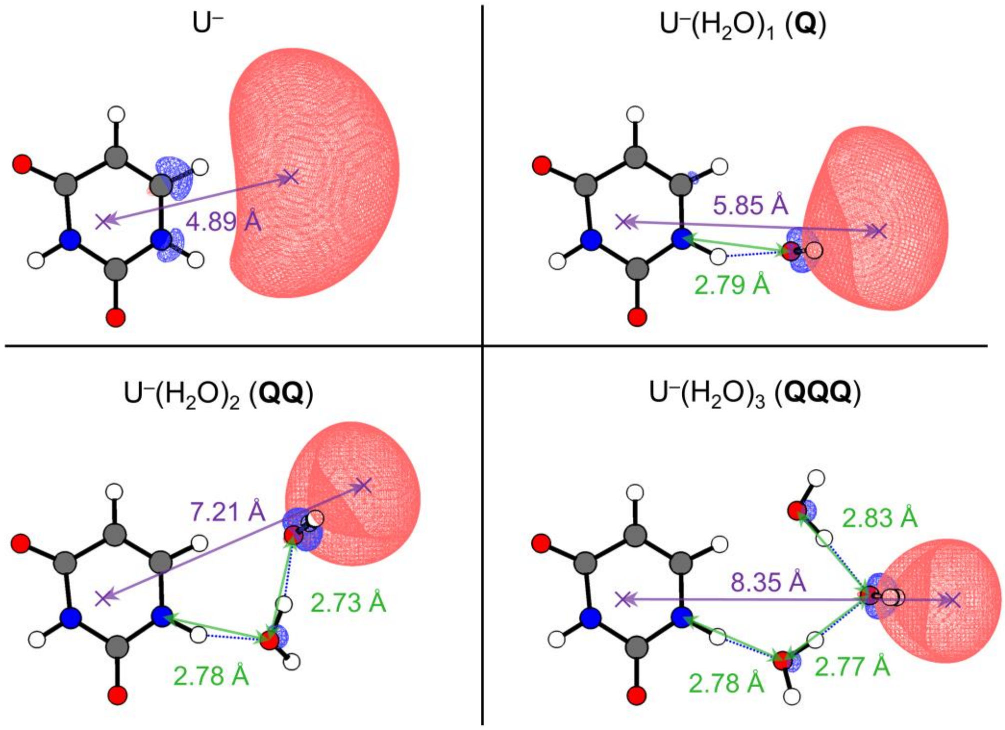

Suppl. Fig. 2. Distances between the centre of mass of U and the excess non-valence electron in the all-Q isomers of $U^-(H_2O)_n$ (purple), and between the donor and acceptor atoms of the intra-cluster hydrogen bonds (green).

Suppl. Note 3. Generation of non-valence states of $U^{-}(H_2O)_n$

The isomeric distributions of cluster anions can be sensitive to the source conditions.[11] In our setup, neutral $U(H_2O)_n$ clusters are first formed in a supersonic expansion. A filament ring ionizer generates electrons that are accelerated into the throat of the expansion, around 10 mm from the face of the nozzle. A plasma is generated with the Ar backing gas, producing low-energy electrons that can attach to $U(H_2O)_n$. This can lead to sole formation of the intact parent cluster anion (1), or evaporation can accompany electron attachment (2):

$$U(H_2O)_n + e^- \rightarrow U^-(H_2O)_n, \qquad (1)$$

$$U(H_2O)_n + e^- \rightarrow U^-(H_2O)_{n-m} + mH_2O. \qquad (2)$$

In both cases, water molecules are clustered to U before the electron is attached. Therefore, the isomeric structure of each initial cluster contains either R- or Q-site water molecules, making these the most probable isomers to observe upon anion formation. Even if some water clustering continued to occur after electron attachment, computations showed that the Q-isomers were significantly more stable than the P-isomers, owing to their propensity for hydrogen-bonding. As the energy barrier that limits Q–P interconversion is expected to be small (due to the diffuse and polarizable nature of the non-valence electron that separates these isomeric sites), we would nonetheless expect efficient restructuring from P- to Q-isomers. Although the (valence state) R-isomers of the uracil-water cluster anions are lower in energy than the Q-isomers, the rate of Q–R interconversion appears to exceed the timescale of the experiment, i.e. hundreds of microseconds. Unfortunately, our experiment is unable to further probe this interconversion rate.

Suppl. Note 4. Optimized structures for non-valence states of $U^{-}(H_2O)_n$

U^{-}

VDE_0 = 68.0 meV

N	0.000000	0.982033	0.000000
N	-1.138595	-1.026251	0.000000
C	-1.228288	0.351434	0.000000
C	0.053635	-1.701847	0.000000
C	1.239675	-1.062506	0.000000
C	1.270419	0.389785	0.000000
O	2.266827	1.093695	0.000000
O	-2.285337	0.953508	0.000000
H	-0.026281	1.994447	0.000000
H	-2.018069	-1.531259	0.000000
H	-0.030110	-2.788217	0.000000
H	2.180054	-1.604272	0.000000

$U^{-}(H_2O)_1$ [Q]

VDE_1 = 223.678 meV; E_a = 0 meV

N	0.004457	0.978682	0.000022
N	-1.150253	-1.023400	-0.000125
C	-1.230400	0.350436	-0.000068
C	0.038950	-1.693881	-0.000061
C	1.233846	-1.063683	0.000048
C	1.271087	0.385575	0.000045
O	2.272411	1.088153	0.000232
O	-2.277150	0.972683	-0.000115
H	-0.020984	1.990674	0.000076
H	-2.012289	-1.579494	-0.000218
H	-0.061790	-2.777957	-0.000089
H	2.171417	-1.609439	0.000125
O	-2.841480	-3.238081	-0.000251
H	-3.124505	-3.762215	-0.767754
H	-3.124501	-3.761246	0.767918

$U^-(H_2O)_1$ [P]

VDE_1 = 254.799 meV; E_a = 314.515 meV

N	-1.795760	-0.797115	0.000000
N	0.000000	0.652869	0.000000
C	-1.371745	0.518923	0.000000
C	0.856280	-0.413212	0.000000
C	0.418427	-1.688988	0.000000
C	-1.007153	-1.954878	0.000000
O	-1.544229	-3.052736	0.000000
O	-2.143995	1.459982	0.000000
H	-2.799014	-0.933531	0.000000
H	0.371277	1.601472	0.000000
H	1.914615	-0.147033	0.000000
H	1.106943	-2.528173	0.000000
O	4.893026	3.787033	0.000000
H	5.036105	2.829499	0.000000
H	3.927128	3.862183	0.000000

$U^-(H_2O)_1$ [R_2, planar constraint on U]

VDE_1 = 98.911 meV

N	-1.795760	-0.797115	0.000000
N	0.000000	0.652869	0.000000
C	-1.371745	0.518923	0.000000
C	0.856280	-0.413212	0.000000
C	0.418427	-1.688988	0.000000
C	-1.007153	-1.954878	0.000000
O	-1.544229	-3.052736	0.000000
O	-2.143995	1.459982	0.000000
H	-2.799014	-0.933531	0.000000
H	0.371277	1.601472	0.000000
H	1.914615	-0.147033	0.000000
H	1.106943	-2.528173	0.000000
O	4.893026	3.787033	0.000000
H	5.036105	2.829499	0.000000
H	3.927128	3.862183	0.000000

$U^{-}(H_2O)_1$ [R_4, planar constraint on U]
VDE_1 = 67.245 meV

N	0.717185	-0.996650	-0.000031
N	1.758098	1.064333	-0.000042
C	1.917211	-0.306032	-0.000076
C	0.535535	1.677958	-0.000048
C	-0.620669	0.981858	-0.000006
C	-0.567433	-0.463683	-0.000009
O	-1.532329	-1.226856	0.000043
O	2.997136	-0.861948	-0.000004
H	0.793343	-2.006817	-0.000004
H	2.611257	1.616598	-0.000016
H	0.567413	2.767068	-0.000011
H	-1.590035	1.471204	0.000050
O	-3.899789	0.221986	0.000101
H	-3.136148	-0.390105	0.000086
H	-4.680811	-0.337788	0.000120

$U^{-}(H_2O)_2$ [QQ]
VDE_2 = 372.369 meV; E_a = 0 meV

N	-2.026889	0.541911	0.007467
N	0.283522	0.543061	-0.002356
C	-0.865872	1.295647	0.009927
C	0.274021	-0.824154	-0.008666
C	-0.871192	-1.542460	-0.009639
C	-2.144788	-0.852570	-0.002807
O	-3.255365	-1.366727	-0.004738
O	-0.882229	2.515562	0.022788
H	-2.891184	1.068550	0.015513
H	1.194271	1.026040	-0.006499
H	1.262357	-1.285806	-0.013096
H	-0.865479	-2.627289	-0.014420
O	2.983869	1.206488	-0.108821
H	3.540059	1.750047	0.459317
H	3.367088	0.300845	-0.067906
O	3.651071	-1.441902	0.025160
H	4.203152	-1.841593	-0.671048
H	4.061524	-1.751738	0.854361

$U^-(H_2O)_2$ [QP]

VDE_2 = 525.836 meV; E_a = 274.500 meV

N	-1.269060	0.850981	-0.000017
N	0.898914	0.047460	0.000090
C	0.083243	1.155167	0.000109
C	0.409012	-1.225823	-0.000004
C	-0.913552	-1.504489	-0.000101
C	-1.866552	-0.413318	-0.000135
O	-3.086707	-0.509825	-0.000196
O	0.488591	2.304017	0.000158
H	-1.894841	1.646565	-0.000025
H	1.923893	0.165688	0.000154
H	1.176488	-1.999558	0.000008
H	-1.286045	-2.523401	-0.000168
O	3.656095	-0.330648	0.000220
H	4.056713	-0.785486	0.763778
H	4.056835	-0.785314	-0.763374
O	5.949624	-4.477342	0.000375
H	5.651942	-3.948609	0.756133
H	5.652037	-3.948662	-0.755458

$U^-(H_2O)_2$ [PP]

VDE_2 = 499.400 meV; E_a = 402.106 meV

N	2.464970	-0.547935	0.274656
N	0.159848	-0.443309	0.218365
C	1.270039	-1.156308	0.615156
C	0.235047	0.747895	-0.445909
C	1.412896	1.322269	-0.768338
C	2.651276	0.663750	-0.402356
O	3.784802	1.063513	-0.627278
O	1.219158	-2.218419	1.207973
H	3.302630	-1.044774	0.551208
H	-0.755035	-0.838231	0.439980
H	-0.732993	1.189981	-0.690305
H	1.460407	2.269244	-1.296648
O	-6.797442	-0.470696	-2.328657
H	-6.272991	-0.208881	-1.544781
H	-6.128353	-0.670668	-2.991199
O	-5.163596	0.268158	-0.151978
H	-4.522543	-0.412468	0.116992
H	-4.595999	1.036205	-0.332910

U⁻$(H_2O)_2$ [QR_1]

VDE_2 = 402.272 meV; E_a = 17.745 meV

N	1.412911	-1.022963	0.000408
N	-0.276500	0.553029	0.000208
C	0.053822	-0.778571	0.000558
C	0.665393	1.542164	-0.000332
C	1.991949	1.289463	-0.000530
C	2.452367	-0.084690	-0.000187
O	3.613915	-0.465878	-0.000369
O	-0.764797	-1.686971	0.000957
H	1.683168	-1.998735	0.000615
H	-1.273744	0.820559	0.000193
H	0.257503	2.552964	-0.000550
H	2.727785	2.086679	-0.000931
O	-3.682789	-1.293487	-0.001408
H	-2.740490	-1.524486	-0.000473
H	-3.682052	-0.325360	-0.000622
O	-2.886467	1.628864	0.000693
H	-3.073785	2.203675	-0.765885
H	-3.073349	2.203821	0.767304

U⁻$(H_2O)_2$ [QR_2]

VDE_2 = 282.095 meV; E_a = 24.577 meV

N	1.106151	0.149188	-0.027395
N	-1.196474	-0.076650	-0.014220
C	0.021894	-0.698462	-0.032445
C	-1.323018	1.284363	0.007727
C	-0.257093	2.111499	0.013276
C	1.080543	1.549099	-0.000838
O	2.127014	2.179412	0.010039
O	0.144402	-1.923174	-0.050569
H	2.019538	-0.306715	-0.032977
H	-2.057456	-0.640522	-0.011101
H	-2.352832	1.638092	0.021169
H	-0.367072	3.190674	0.031319
O	2.841203	-2.248437	-0.042233
H	1.867396	-2.382333	-0.044789
H	3.160142	-2.670618	0.760883
O	-3.848753	-0.900024	0.026869
H	-4.462457	-0.834081	-0.724259
H	-4.419879	-0.843469	0.811884

$U^{-}(H_2O)_2$ [QR_3]

VDE_2 = 269.510 meV; E_a = 25.437 meV

N	0.875493	-0.419368	-0.036143
N	-1.420438	-0.106327	-0.010329
C	-0.379312	-1.010200	-0.027581
C	-1.228158	1.240437	0.002639
C	0.003374	1.800505	-0.001329
C	1.159830	0.939050	-0.018348
O	2.334555	1.325664	-0.016562
O	-0.531971	-2.216578	-0.033624
H	1.675458	-1.053637	-0.044353
H	-2.388890	-0.449949	-0.000043
H	-2.146641	1.825772	0.017621
H	0.148632	2.875219	0.009894
O	3.774273	-0.965116	-0.028662
H	4.260220	-1.094167	0.791143
H	3.464194	-0.030563	-0.013372
O	-4.204596	-0.181784	0.042593
H	-4.778203	0.052330	-0.706352
H	-4.732644	0.038616	0.828528

$U^{-}(H_2O)_2$ [QR_4]

VDE_2 = 270.343 meV; E_a = 102.867 meV

N	-0.247766	-1.414166	-0.004310
N	1.481168	0.120216	-0.007771
C	1.125482	-1.208333	0.014582
C	0.565233	1.128473	-0.047076
C	-0.769908	0.908524	-0.066023
C	-1.248887	-0.452598	-0.040596
O	-2.428274	-0.813126	-0.046834
O	1.916242	-2.131575	0.048314
H	-0.544156	-2.382270	0.012097
H	2.476955	0.380546	0.009134
H	1.002603	2.125532	-0.061427
H	-1.492239	1.717816	-0.098257
O	-4.311116	1.230319	-0.013254
H	-4.682996	1.203573	0.873161
H	-3.693250	0.470835	-0.047810
O	3.789462	1.629379	0.029692
H	4.235297	1.995949	0.812612
H	4.301938	1.969290	-0.723612

$U^{-}(H_2O)_3$ [QQQ]

VDE_3 = 516.606 meV; E_a = 0 meV

N	-2.549300	0.559077	-0.028234
N	-0.237890	0.547401	0.025052
C	-1.383290	1.304739	-0.011462
C	-0.253964	-0.819291	0.038766
C	-1.406220	-1.529013	0.021141
C	-2.674971	-0.834322	-0.013854
O	-3.789425	-1.342313	-0.031676
O	-1.393039	2.525428	-0.028837
H	-3.409746	1.091225	-0.054708
H	0.666264	1.045120	0.045021
H	0.728207	-1.296315	0.061706
H	-1.406745	-2.613749	0.031766
O	2.310581	1.664215	0.134552
H	2.539874	2.569910	-0.096664
H	3.136050	1.141685	0.042694
O	4.521946	0.017858	-0.103640
H	5.034995	0.069850	-0.935660
H	5.203945	0.073809	0.595294
O	2.695886	-2.135986	0.053035
H	3.184056	-2.957264	-0.065052
H	3.376508	-1.435919	0.002852

$U^{-}(H_2O)_3$ [QQR_3]

VDE_3 = 412.209 meV; E_a = –80.750 meV

N	-1.488717	0.392186	-0.028083
N	0.824094	0.523806	-0.022794
C	-0.369642	1.209404	-0.015544
C	0.897152	-0.836929	-0.028161
C	-0.207613	-1.619535	-0.034808
C	-1.506835	-0.996408	-0.034157
O	-2.587601	-1.598811	-0.036377
O	-0.449720	2.424784	0.002745
H	-2.394847	0.861622	-0.020332
H	1.705309	1.060203	-0.018184
H	1.910348	-1.241781	-0.025191
H	-0.144000	-2.702180	-0.037881
O	3.478071	1.337134	-0.097042
H	3.990518	1.916103	0.477276
H	3.910010	0.454373	-0.039120
O	4.273490	-1.271389	0.074585
H	4.863956	-1.651862	-0.600988
H	4.672057	-1.556595	0.918791
O	-4.445575	0.361977	0.000879
H	-4.938343	0.369533	0.826751
H	-3.950343	-0.490124	-0.007285

$U^{-}(H_2O)_3$ [QR_2R_4]

VDE_3 = 306.934 meV; E_a = 23.938 meV

N	0.202901	0.955152	0.007690
N	-1.357230	-0.753996	0.005424
C	-1.128350	0.593928	0.027874
C	-0.343925	-1.667650	-0.034539
C	0.958786	-1.308923	-0.054773
C	1.294460	0.096564	-0.034967
O	2.432588	0.567559	-0.052887
O	-2.031014	1.427390	0.062470
H	0.383622	1.961227	0.015414
H	-2.325261	-1.110464	0.016613
H	-0.678566	-2.703559	-0.048947
H	1.762842	-2.037304	-0.086556
O	4.474936	-1.310321	-0.024314
H	4.911870	-1.196085	0.824665
H	3.801830	-0.598773	-0.056874
O	-3.540812	-2.417008	0.005228
H	-3.992359	-2.811463	0.771538
H	-4.013046	-2.773631	-0.767245
O	-0.626941	3.755049	0.066705
H	-1.347915	3.087967	0.065038
H	-0.778595	4.30914	-0.704632

$U^{-}(H_2O)_3$ [PPP]

VDE_3 = 771.694 meV; E_a = 424.427 meV

N	3.340824	0.094827	0.575824
N	1.034782	0.081588	0.470826
C	2.149494	0.210300	1.270330
C	1.102775	-0.138318	-0.874968
C	2.276872	-0.247538	-1.531838
C	3.519475	-0.129315	-0.794770
O	4.650236	-0.206264	-1.253912
O	2.106073	0.406430	2.470903
H	4.181620	0.184281	1.132652
H	0.119613	0.156410	0.919726
H	0.130486	-0.216951	-1.365966
H	2.319108	-0.422410	-2.602141
O	-5.408269	-2.545355	0.271735
H	-5.039870	-1.654824	0.119452
H	-4.625574	-3.095197	0.383480
O	-4.074089	-0.025792	-0.173568
H	-3.408365	0.078927	0.534962
H	-3.512858	-0.135673	-0.964511
O	-5.443258	2.49898	-0.44722
H	-4.668604	3.069131	-0.49465
H	-5.062029	1.606626	-0.345586

Supplementary References

1. Anstöter, C. S. & Matsika, S. Understanding the Interplay between the Nonvalence and Valence States of the Uracil Anion upon Monohydration. *J. Phys. Chem. A* **124**, 9237–9243 (2020).
2. Kendall, R. A., Dunning, T. H., Jr. & Harrison, R. J. Electron affinities of the first-row atoms revisited. Systematic basis sets and wave functions. *J. Chem. Phys.* **96**, 6796–6806 (1992).
3. Yanai, T., Tew, D. P. & Handy, N. C. A new hybrid exchange–correlation functional using the Coulomb-attenuating method (CAM-B3LYP). *Chem. Phys. Lett.* **393**, 51–57 (2004).
4. M. J. Frisch et al. Gaussian 16, Revision C.01. Gaussian, Inc. (2016).
5. Raghavachari, K., Trucks, G. W., Pople, J. A. & Head-Gordon, M. A fifth-order perturbation comparison of electron correlation theories. *Chem. Phys. Lett.* **157**, 479–483 (1989).
6. Bartlett, R. J., Watts, J. D., Kucharski, S. A. & Noga, J. Non-iterative fifth-order triple and quadruple excitation energy corrections in correlated methods. *Chem. Phys. Lett.* **165**, 513–522 (1990).
7. Clarke, C. J., Burrow, E. M. & Verlet, J. R. R. The valence electron affinity of uracil determined by anion cluster photoelectron spectroscopy. *Phys. Chem. Chem. Phys.* **26**, 20037–20045 (2024).
8. Castleman, A. W. & Bowen, K. H. Clusters: Structure, Energetics, and Dynamics of Intermediate States of Matter. *J. Phys. Chem.* **100**, 12911–12944 (1996).
9. Shin, J.-W., Hammer, N. I., Headrick, J. M. & Johnson, M. A. Preparation and photoelectron spectrum of the 'missing' (H2O)4- cluster. *Chem. Phys. Lett.* **399**, 349–353 (2004).

10. Hammer, N. I., Roscioli, J. R. & Johnson, M. A. Identification of Two Distinct Electron Binding Motifs in the Anionic Water Clusters: A Vibrational Spectroscopic Study of the (H2O)6- Isomers. *J. Phys. Chem. A* **109**, 7896–7901 (2005).

11. Lietard, A. & Verlet, J. R. R. Selectivity in Electron Attachment to Water Clusters. *J. Phys. Chem. Lett.* **10**, 1180–1184 (2019).
